# Intra-articular injection of mono-iodoacetate induces osteoarthritis of the hip in rats

**DOI:** 10.1186/s12891-016-0985-z

**Published:** 2016-03-18

**Authors:** Shuichi Miyamoto, Junichi Nakamura, Seiji Ohtori, Sumihisa Orita, Takanori Omae, Takayuki Nakajima, Takane Suzuki, Kazuhisa Takahashi

**Affiliations:** Department of Orthopaedic Surgery, Graduate School of Medicine, Chiba University, 1-8-1 Inohana, Chuo-ku, Chiba City, Chiba 260-8677 Japan

**Keywords:** Intra-articular injection, Mono-iodoacetate, Osteoarthritis, Hip in rats

## Abstract

**Background:**

The mechanism for hip pain has been unclear because of a lack of experimental animal models. We aimed to establish an intra-articular injection technique to the rat hip and to document the effect of intra-articular mono-iodoacetate (MIA) injection to the rat hip with radiography and histology.

**Methods:**

Using 60 6-week-old male Sprague Dawley rats, 25 μl of sterile saline (control group; *n* = 30) and 25 μl of sterile saline with 2 mg of MIA (MIA group; *n* = 30) was injected into the right hip joints via posterior approach using a 27G needle. The animals were examined with X-ray and histology 7, 14, 28, 42, and 56 days later (MIA group [*n* = 6] and control group [*n* = 6], respectively).

**Results:**

The MIA group showed progressive radiographic changes to the hip joint during the experimental period, whereas the control group maintained a normal appearance. The microanatomic appearance was consistent with X-ray images of progressive destruction in the MIA group and normal tissue in the control group. Osteoarthritic (OA) changes became apparent at 42 and 56 days in the MIA group.

**Conclusions:**

We established an intra-articular injection technique to the rat hip, creating a hip OA model in the rat by intra-articular injection of MIA.

## Background

Osteoarthritis (OA) is the most common arthritis in the elderly population [1]. Clinical signs of hip OA are groin pain, restricted range of motion, and gait disturbance [2, 3], which deteriorates the quality of life. It is essential to understand how histologic changes correlate with the onset of pain and joint destruction. OA is characterized by a progressive degeneration of articular cartilage [4, 5]. Increases of mechanical stress and biochemical factors in the affected joints are thought to be responsible for the pathogenesis of OA. It has been suggested that tumor necrosis factor α, interleukin-6, and calcitonin gene-related peptide are up-regulated in OA [6, 7]. However, the mechanism for hip pain has been unclear because of a lack of experimental animal models. For knee OA, several animal models have been proposed, such as the anterior cruciate ligament transection model [8], partial medial meniscectomy model [9], or a combination of both [10]. Knee OA can be chemically induced by intra-articular injection of a metabolic inhibitor, mono-iodoacetate (MIA) [11]. This compound inhibits the production of glyceraldehyde-3-phosphate dehydrogenase by articular chondrocytes, leading to disruption of glycolytic energy metabolism and synthetic processes, and eventually to cell death [12]. In knee MIA models using rat and rabbit, cartilage lesions with loss of proteoglycan matrix and chondrocyte death were induced in the early phases of cartilage degeneration. With progressive cartilage deterioration, joint destruction with exposed subchondral bone, pain and functional impairment were produced similar to human OA [13]. However, to the best of our knowledge, intra-articular MIA injection to the rat hip has not been reported, partly because intra-articular injection to the rat hip itself is technically demanding.

OA is diagnosed with radiographic findings [4]. Recently an X-ray imaging unit for animal study has been introduced as in vivo imager (Xtreme, Bruker, WI) [14]. This device is useful to evaluate morphological changes of bone noninvasively without sacrificing the animal.

The purpose of this study was to establish an intra-articular injection technique to the rat hip and to document the effect of intra-articular MIA injection to the rat hip using radiography and histology.

## Methods

All protocols for animal procedures were reviewed and approved by the ethics committee of our institution and followed the National Institutes of Health Guidelines for the Care and Use of Laboratory Animals.

### Intra-articular injection technique to rat hip

**Fig. 1:**
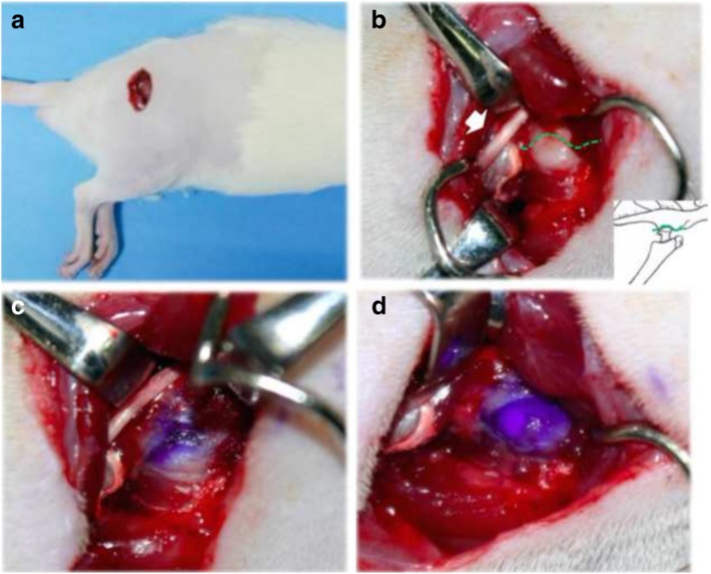
Intra-articular injection technique to rat hip. **a** The right hip is exposed via posterior approach in the lateral decubitus position. **b** The posterior aspect of the capsule, the joint space (*broken line*), and the sciatic nerve (*white arrow*) are identified. **c** Intra-articular injection of 25 μl of Pyoktanin Blue. **d** Leak test of the pigment

To confirm the validity of intra-articular injection into the hip, three male Sprague-Dawley (SD) rats weighing 200–300 g at 6 weeks of age were prepared (CLEA, Tokyo, Japan). All rats were anesthetized with an intraperitoneal injection of sodium pentobarbital (40 mg/kg) and treated aseptically throughout the experiments. A posterior approach was used to expose the right hip in the lateral decubitus position. After removal of the hair, a 1.5–2.0 cm skin incision was made in the direction of the posterior line of the greater trochanter with the hip in neutral position (Fig. 1a). The superficial gluteus muscle was identified and resected. Deeper than that, the obturator internus and externus muscles, and the sciatic nerve, were identified. The obturator intermus and externus muscles were detached from the greater trochanter. The posterior aspect of the capsule was exposed, protecting the sciatic nerve. The joint space was identified by passive motion of the limb (Fig. 1b). Then 25 μl of 1 % Pyoktanin Blue solution (Kishida Chemical Company, Osaka) was injected into the joint using a 27G needle (Fig. 1c). We confirmed that the pigment was not leaked and stayed within the joint in all three rats (Fig. 1d). Thus we accepted this intra-articular injection technique to the rat hip joint for further study. The superficial gluteus muscle was sutured and the skin was closed.

### Rat MIA hip model

Thirty male SD rats were treated with a single intra-articular injection of 2 mg of MIA (Sigma-Aldrich, St. Louis, MO) in 25 μl of sterile saline (MIA group) and 30 were treated only with 25 μl of sterile saline (control group) in their right hips. The doses of MIA were based on previous literature that showed destruction of articular cartilage [15, 16]. The animals were examined 7, 14, 28, 42 and 56 days later (MIA group [*n* = 6] and control group [*n* = 6], respectively).

### X-ray imaging of the hip joint

After intraperitoneal anesthesia, the rats were laid in the supine position with 0° of hip flexion, abduction, and internal and external rotation. Both limbs were fixed on the table with tape. Anteroposterior bilateral X-rays of the hips were taken with an in vivo imager (Xtreme, Bruker, WI) [14]. Lateral images also were taken in 45° of flexion and abduction and 0° of internal and external rotation. X-ray examination was performed 7, 14, 28, 42 and 56 days later (MIA group [*n* = 6] and control group [*n* = 6], respectively).

X-ray images were classified based on the Kellgren and Lawrence (KL) system [4] as grade 0 (none: no radiographic features of OA), grade 1 (doubtful: doubtful joint space narrowing [JSN] and possible osteophyte), grade 2 (minimal: the presence of definite osteophytes and possible JSN), grade 3 (moderate: multiple osteophytes, definite JSN, sclerosis, possible bony deformity), and grade 4 (severe: large osteophytes, marked JSN, severe sclerosis and definite bony deformity).

### Tissue preparation

The rats then were perfused transcardially with 0.9 % saline, followed by 500 ml of 4 % paraformaldehyde in phosphate buffer fixative (0.1 M, pH 7.4). The soft tissues around the right hip joint including cartilage, synovium and capsule were resected. The resected limbs were cut at midfemur and the center of the femoral head immersed in buffered paraformaldehyde fixative at 4 °C for 1 week. The specimens were continuously demineralized in reagent K-CX (FALMA, Tokyo, Japan) for 30 h and 5 % sodium sulphate for 16 h, followed by paraffin embedding for subsequent coronal sectioning. The samples were serially sectioned in steps of 8 μm, and stained using hematoxylin and eosin (H-E), Safranin O, and Toluidine Blue, respectively.

### Histopathological analysis

Specimens were randomly assigned identification numbers to blind investigators to group association, and assessed by light microscopy. Semi-quantitative histopathological grading was performed according to a modified Mankin scoring system established for grading OA changes [17]. Mankin scores normally consider five characteristics: structure, chondrocyte number, chondrocyte clustering, proteoglycan content, and subchondral bone plate and/or tidemark changes, including vascular invasion into cartilage. Three sections from each sample were scored by two different blinded observers, for a maximum possible score of 14. Total points were classified as low (1–3 points), moderate (4–7 points), or high (>8 points). Osteophytes were defined as outgrowths of the bone and cartilage occurring at the joint margins. Osteophytic regions were examined in sections stained with either HE, Safranin O, or Toluidine blue stains. To evaluate the incidence of osteophyte formation, total osteophyte number from five sections 100 μm apart was evaluated from each hip joint. The percent incidence of osteophytes was calculated for each group. Images of articular cartilage and subchondral bone were examined using an Olympus fluorescence microscope (DP71, Japan) with 4× objective lens.

### Statistical analysis

Statistical significance between MIA and control groups was calculated using one- way analysis of variance and Fisher’s exact probability tests (SPSS 16.0 Chicago, IL, USA). Differences were considered significant for *P* values <0.05.

## Results

### X-ray findings

**Fig. 2:**
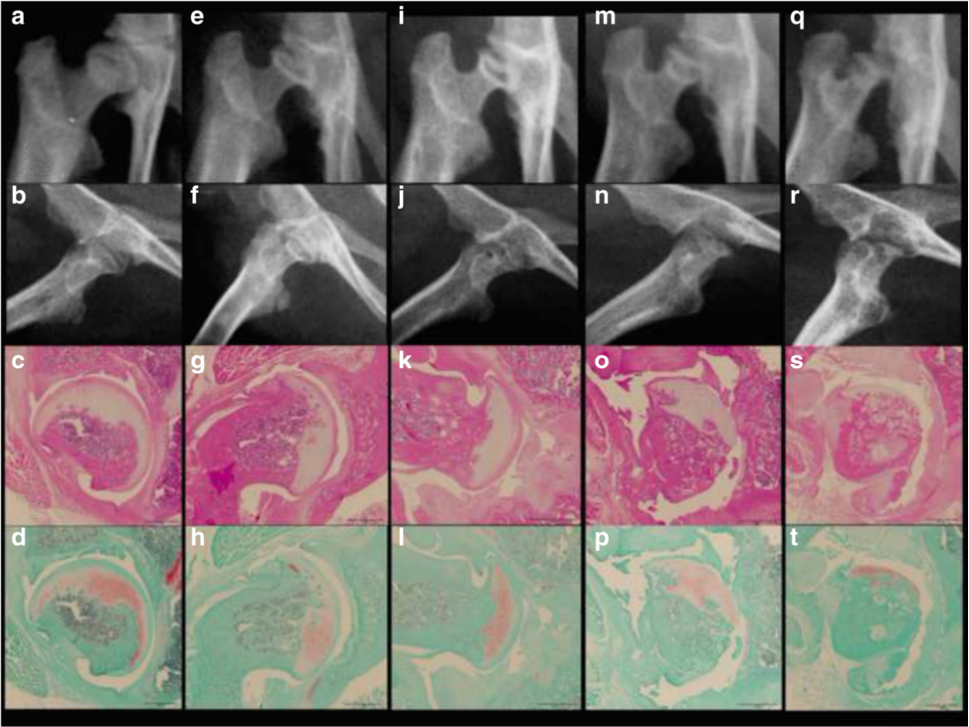
Time course of X-ray and histopathology of the hip in MIA group. After 7 days (**a**–**d**), 14 days (**e**–**h**), 28 days (**i**–**l**), 42 days (**m**–**p**), and 56 days (**q**–**t**). Anteroposterior (**a**, **e**, **i**, **m**, and **q**) and lateral (**b**, **f**, **j**, **n**, and **r**) radiographs of the rat hip. Hematoxylin and eosin (**c**, **g**, **k**, **o**, and **s**) and Safranin O (**d**, **h**, **l**, **p**, **t**) staining. Scale bars are 1 mm. This shows no evidence of narrowing of the joint space (**a**, **b**) or degeneration (**c**, **d**) at 7 days, but possible joint space narrowing (**e**, **f**) and extensive involvement of cartilage (**g**, **h**) at 14 days, definite joint space narrowing and deformity of the femoral head (**i**, **j**) and multifocal collapse of bony trabeculae in the acetabulum and the femoral head (**k**, **l**) at 28 days, severely deformed femoral head and erosive acetabulum (**m**, **n**) and peeling of articular cartilage and exposure of subchondral bone (**o**, **p**) at 42 days, and destruction of the femoral head and acetabulum (**q**, **r**) and complete disappearance of articular cartilage (**s**, **t**) at 56 days

**Fig. 3:**
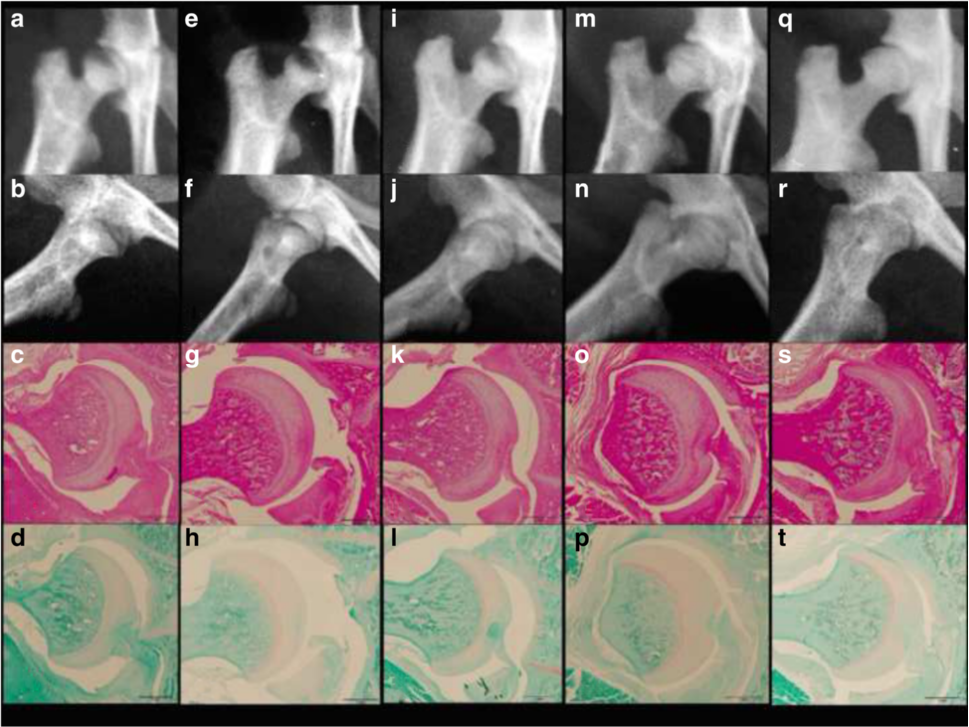
Time course of X-ray and histopathology of the hip in the control group. After 7 days (**a**–**d**), 14 days (**e**–**h**), 28 days (**i**–**l**), 42 days (**m**–**p**), and 56 days (**q**–**t**). Anteroposterior (**a**, **e**, **i**, **m**, and **q**) and lateral (**b**, **f**, **j**, **n**, and **r**) radiographs of the rat hip. Hematoxylin and eosin (**c**, **g**, **k**, **o**, and **s**) and Safranin O (**d**, **h**, **l**, **p**, **t**) staining. Scale bars are 1 mm. This shows no evidence of degeneration or narrowing of the joint space during the period

The MIA group showed progressive changes to the hip joint during the experimental period (Fig. 2), whereas the control group maintained a normal appearance (Fig. 3). Seven days post-injection, X-rays showed no evidence of degeneration or narrowing of the joint space in either MIA (Fig. 2a, b) or control groups (Fig. 3a, b). Thus all hips were grade 0 in KL classification. By 14 days, the MIA group showed possible JSN and cystic changes in the femoral head; one hip was grade 1 and five hips were grade 2 in KL classification (Fig. 2e, f). By 28 days, the MIA group showed definite JSN and deformity of the femoral head, evidenced by a flattened epiphysis. The articular surface was irregular and incongruent; two hips were grade 2 and four hips were grade 3 in KL classification (Fig. 2i, j). By 42 days, the MIA group showed severely deformed femoral heads, bone atrophy and erosion in the acetabulum. The articular surface became incongruent but the joint space was maintained. One hip was grade 3 and five hips were grade 4 in KL classification (Fig. 2m, n). By 56 days, the MIA group showed destructive changes of the femoral head and subchondral bone of the acetabulum. All six hips were grade 4 in KL classification (Fig. 2q, r).

**Fig. 4:**
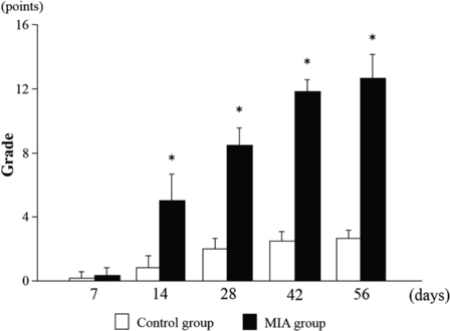
Mankin scores in the MIA and control groups. Osteoarthritic changes become progressive in the MIA group in comparison with controls after 14, 28, 42 and 56 days, respectively. *p <0.05

### Histopathological findings

Microanatomic appearance was consistent with X-ray images with progressive destruction in the MIA group (Fig. 2) and normal tissue in the control group (Fig. 3). By day 7 post-injection, the MIA group showed an expanded synovial membrane by edema mixed with mild to moderate infiltrates of lymphocytes, macrophages and plasma cells (Fig. 2c, d). However, chondrocyte degeneration was absent both in the acetabulum and the femoral head. The femoral head was spherical and the subchondral bone was not involved. The mean Mankin score was (Fig. 4) 0.3 (range, 0–1) in the MIA group and 0.2 (range, 0–1) in the control group, without a significant difference (*p* = 0.549). The incidence of osteophytes was 0 % in both groups.

By 14 days, extensive collapse of the cartilaginous matrix with marked loss of chondrocytes was noted in the MIA group (Fig. 2g, h). The articular surface of the femoral head became irregular. Focal areas of the subchondral bone marrow were replaced by loosely arranged spindle cells contained within a fine stroma. These areas of bone marrow replacement were marked by the formation of cysts which were present underneath damaged cartilage of the femoral head and were sharply demarcated from normal trabecular bone and marrow elements. The mean Mankin score (Fig. 4) was 5.0 (range, 4–8) in the MIA group and 0.8 (range, 0–2) in the control group (*p* = 0.0002). The incidence of osteophytes was one of six hips (17 %) in the MIA group and none in the control group.

By 28 days, multifocal collapse of bony trabeculae in the acetabulum and the femoral head were evident in the MIA group (Fig. 2k, l). Osteophyte formation led to capital drop of the flattened femoral head. Fragmented bone was surrounded by numerous osteoclasts and was contained within focally extensive areas of fibrosis that replaced adjacent bone trabeculae and marrow elements. Small amounts of cellular debris also were present in these areas. Gross lesions consisted of a well-demarcated area of cartilage erosion both in the acetabulum and the femoral head. The mean Mankin score (Fig. 4) was 8.5 (range, 7–10) in the MIA group and 2.0 (range, 1–3) in the control group (*p* = 0.0001). The incidence of osteophytes was two of six hips (33 %) in the MIA group and none in the control group.

By 42 days, extensive areas of articular cartilage were shed and disappeared in the MIA group (Fig. 2o, p). Chondrocytes were shrunken with marked loss of chondrocyte cellular detail. There was multifocal collapse and fragmentation of bony trabeculae, surrounded by epiphyseal chondrocytes. The mean Mankin score (Fig. 4) was 11.8 (range, 11–13) in the MIA group and 2.5 (range, 2–3) in the control group (*p* = 0.0001). The incidence of osteophytes was three of six hips (50 %) in the MIA group and none in the control group.

By 56 days, articular cartilage had disappeared completely in the MIA group (Fig. 2s, t). Chondrocytes were fragmented and necrotic, and exposed to the articular surface with a marked loss of height. Subchondral trabecular bone was partially exposed to the articular surface. Bone remodeling of the trabecular bone was present with bone resorption and active osteoclastic activity. Cystic structures demarcated by fibrous tissue and containing small amounts of necrotic debris were present. The mean Mankin score (Fig. 4) was 12.7 (range, 11–14) in the MIA group and 2.7 (range, 2–3) in the control group (*p* = 0.0001). The incidence of osteophytes was three of six hips (50 %) in the MIA group and none in the control group.

## Discussion

This is the first study of a rat hip OA model induced by intra-articular injection of MIA. The histological appearance of MIA-injected hips showed an OA-like appearance. The current study outlines the time-dependent progression of histologic lesions in the rat hip with major emphasis on subchondral bone as well as radiographic changes. The intra-articular injection technique to the rat hip using 25 μl of solution injected into the joint using a 27G needle via a posterior approach in the lateral decubitus position was shown to be reliable.

The time course for development of osteoarthritis after MIA injection is of great interest. In our rat hip OA model, articular cartilage degeneration became evident 14 days after MIA injection. By comparison, the intra-articular MIA injection in the rat knee produced OA changes within 7 days post-MIA [9, 18]. Guzman et al. [18] had already observed extensive areas of chondrocyte degeneration at 1 day and moderate collapse of the cartilaginous matrix with marked loss of chondrocyte by 5 or 7 days. Orita et al. [16] also reported OA change after 7 days. In the hip, we found that progression of the joint destruction continued through 56 days post-MIA. Ferreira-Gomes et al. reported time-dependent histological changes in the rat knee and a dose-dependent response in the MIA model [19]. In that study, there was a marked decrease of the thickness of the articular cartilage by 31 days with 0.3 mg of MIA; subchondral bone became exposed with 1 mg; and thickening of the subchondral bone was apparent with 2 mg [19]. In the current study, such OA changes became apparent by 42 and 56 days with 2 mg of MIA injection into the rat hip. It should be investigated whether time to cartilage degeneration is different between the hip and the knee.

Radiological evaluation is a useful technique in animal studies to analyze a joint lesion noninvasively and repeatedly. We succeeded in visualizing the anteroposterior and lateral views of the rat hip with an in vivo imager, as one would for human evaluations. In the current study, X-rays showed a time-dependent progression of deformity of the femoral head and the acetabulum consistent with histopathology.

To our best knowledge, radiographic monitoring of progressive osteoarthritis of the rat hip in addition to histology has not been reported. We suggest that using radiologic evaluation can reduce the unnecessary sacrifice of animals.

The cause and source of pain is uncertain in OA. Sensory innervation of the hip in rats is distributed by the L2, L3 and L4 dorsal root ganglia [20]. A novel rat model of hip pain with intra-articular administration of nerve growth factor produced inflammatory arthritis and pain [21]. Animal models are essential to clarify the source of hip pain. We believe that our rat MIA hip model will contribute in the future to studies of the source of the pain in hip OA.

There are several limitations to this study. First, it should be confirmed which type of OA was induced in the rat MIA hip model. MIA has been widely accepted to induce OA in animal models [9, 16, 18]. This model demonstrated characteristics of inflammatory arthritis that was progressive. It involved rapidly destructive coxarthritis rather than chronic OA secondary to acetabular dysplasia with insufficient acetabular coverage. Second, JSN based on anteroposterior radiographs was not precise because the radiographs were taken in the supine position without weight bearing. By definition, the KL classification [4] is based on anteroposterior radiographs during weight-bearing. This is difficult to accomplish in an animal model. Thus we confirmed cartilage degeneration and JSN using histopathology. Third, we did not study the dose-dependent response of MIA in the rat hip as we only used a single 2 mg dose of MIA.

Effects of 0.3, 1 mg, or more of MIA, based on doses used in previous studies, should also be investigated. Fourth, young rats at 6 weeks of age instead of old were applied because of relatively long-term study for rat until 56 days and because of adjustment to Orita’s study of knee MIA model [16]. It is of great interest whether effect of MIA shows age dependent progression or not. Fifth, this study lacks analysis for pain transmitting substances in the local tissues or dorsal root ganglia [20]. The level of inflammatory cytokines and their receptors, as well as macrophage and endothelial cell markers, should be investigated quantitatively using protein determination or gene expression. Sixth, behavioral testing is an essential part of pain research [22, 23]. Further research employing functional evaluations such as gait analysis are needed. Seventh, quantitative assessment of bone remodeling using micro-computed tomography, OA biomarkers, synovial inflammation, immunohistochemical staining for cartilage specific proteins, and cell death has not been investigated yet, but they should be clarified for characteristics of rat MIA hip model in the future.

## Conclusion

In conclusion, we established an intra-articular injection technique to the rat hip using MIA that consistently causes progressive hip OA.

## Abbreviation

MIA: mono-iodoacetate
OA: osteoarthritis
JSN: joint space narrowing
